# Leniolisib: a drug providing a promising avenue for the treatment of activated phosphoinositide 3-kinase δ syndrome (APDS)

**DOI:** 10.1097/MS9.0000000000002189

**Published:** 2024-05-20

**Authors:** Anoshia Ali, Amsal Qureshi, Anum Fatima Shigri, Areesha Moiz Alvi, Abdullah Malikzai

**Affiliations:** a Agha Khan University hospital (AKU); b Agha Khan University hospital (AKU) Karachi, Pakistan; c Kabul Medical University, Kabul, Afghanistan

Activated phosphoinositide 3-kinase δ syndrome (APDS) (OMIM 615513) is an inborn error of immunity inherited in an autosomal dominant pattern. It was first reported in 2013 with over 285 patients identified thus far^1,3^. Phosphatidyl-inositol-3kinases (PI3Ks) are a group of enzymes encoded by the PIK3 genes involved in a wide spectrum of biological functions. APDS is the result of mutation in these genes either due to a gain-of-function mutation in the PIK3CD gene, which encodes p110δ (APDS1) or loss-of-function mutation in PIK3R1 gene, which encodes p85α (APDS2)^2^. This leads to immunosuppression, affecting both humoral and cell-mediated immunity. The syndrome has a diverse presentation, which includes recurrent bacterial as well as viral respiratory tract infections, gastrointestinal disease, non-malignant and malignant lymphoproliferation, autoimmune cytopenias autoimmune thyroiditis, and susceptibility to EBV^1,3^.

Laboratory findings in patients with APDS usually include dysgammaglobulineamia and increased IgM levels. Decreased response to polysaccharide vaccines has also been reported in patients with APDS^1^.

Thus far the management of APDS has been empirical. This includes immunomodulatory therapies, prophylactic antimicrobials, immunoglobulin replacement therapy (IRT), and surgical procedures including splenectomies, otosinopulmonary surgeries, and hematopoietic stem cell transplantation^4^. However, none of these treatments target the pathogenesis of the disease itself.

On 23 March 2023, the Food and Drug Administration (FDA) licensed a new oral drug named Joenja (Leniolisib) for the treatment of APDS in adults and pediatric patients (≥12 years old). It is a potent, selective PI3K delta inhibitor. It inhibits the signaling pathways that cause dysregulation of B and T immune cells. It reduces PI3Kδ pathway hyperactivity, partially reconstitutes lymphocyte subsets, and decreases lymphoproliferation^4^.

Thirty-one patients with Activated phosphoinositide 3-kinase δ syndrome aged older than or equal to 12 years took part in an international, randomized, phase 3, triple-blinded trial to receive 70 mg Leniolisib or placebo daily (x2) for 12 weeks. First a non-randomized, open-label trial with 6 participants that had APDS was carried out to determine a titrating dose. This was followed by a randomized, triple-blinded placebo-controlled, fixed-dose trial with 31 participants having APDS/PASLI. On day 1, patients were randomized 2:1 to receive 70 mg Leniolisib or placebo orally every 12 h twice daily. Efficacy and safety assessments were made on days 15, 29, 57, and 85, with pharmacokinetic (PK) assessments on days 29, 57, and 85, respectively. The results of the trial stated that Leniolisib significantly reduced lymphadenopathy as 26% of patients in the Leniolisib group (*n*=19) achieved complete absence of index lymphadenopathy, whereas the other 74% achieved a partial response. In comparison, the placebo group (*n*=9), 45% achieved partial response, 44% had stable disease, and 11% had an unknown response. Leniolisib also decreased splenomegaly compared to the placebo. In the Leniolisib group (*n*=13), 38% achieved complete response, 54% achieved partial response, and 8% had stable disease at 12 weeks. In the placebo group (*n*=5), 20% achieved complete response, while the remaining 80% experienced worsening disease. The Leniolisib group also showed improvement in immunodeficiency. It significantly increased the percentage of naïve B cells, improved key lymphocyte subsets, and greatly reduced serum IgM. The mean IgM level decreased 208.26 mg/dl from baseline by day 85 in the Leniolisib and 10.00 mg/dl in the placebo arm. General assessment scores also reflected clinically meaningful improvements for the leniolisib group^4^.

Furthermore, another clinical trial, 12 weeks open-label, multisite, within-subject, dose-escalation study of oral leniolisib, consisting of 6 APDS patients was conducted in order to assess the safety, pharmacokinetics, and effects of the said drug. After completion of the trial the patients showed decrease in lymph node size and spleen volume by 39% and 40%, respectively. Post trail the recommended dosage set by FDA is 70 mg (oral) twice daily, twelve hours apart, with or without food, in all patients older than or equal to 12 years of age, weighing greater than or equal to 45 kg.

So far, the adverse effects seen with use of Leniolisib are primarily grade 1 (headache, sinusitis) based on the criteria for adverse events (CTCAE) set by US Department of Health and Human Services, with many already present at baseline^4^. No significant clinical or laboratory toxicities or side effects have been noted. Recent reports suggest that PI3Kδ inhibition can result in genomic instability. However, this effect in APDS patients taking Leniolisib remains unknown. Long-term monitoring and surveillance for lymphoma is required with the use of leniolisib like other PI3Kδ inhibitors^5^. The pregnancy status of a patient also needs to be verified before the initiation of Leniolisib due to its potential Embryo-Fetal toxicity.

In conclusion, considering the currently available therapeutics for APDS, Joenja (Leniolisib) is a promising drug for targeted therapy of the disease itself and is the first of its kind that has been approved thus far. However, subsequent trials should be carried out to determine the long-term effects of the drug in APDS patients and well as its usage in the younger pediatric population needs to be investigated.

## Ethical approval

Not applicable.

## Consent

Informed consent was not required for this Editorial Article

## Source of funding

This letter was not funded by any organization or institution.

## Author contribution

All authors contributed equally.

## Conflicts of interest disclosure

The author of this article has no affiliation with or involvement in any organization or entity with any financial or non-financial interest in the subject matter or materials discussed in this letter.

## Research registration unique identifying number (UIN)

Not applicable.

## Guarantor

Abdullah Malikzai.

## Data availability statement

Not applicable.

## Provenance and peer review

Not applicable.
